# Anemia prevalence and associated factors among school-children of Kersa Woreda in eastern Ethiopia: A cross-sectional study

**DOI:** 10.1371/journal.pone.0283421

**Published:** 2023-03-24

**Authors:** Kabtamu Gemechu, Haftu Asmerom, Lealem Gedefaw, Mesay Arkew, Tilahun Bete, Wondimagegn Adissu

**Affiliations:** 1 School of Medical Laboratory Science, College of Health and Medical Science, Haramaya University, Harar, Ethiopia; 2 Faculty of Health Science, School of Medical Laboratory Science, Institute of Health, Jimma University, Jimma, Ethiopia; 3 Department of Psychiatry, School of Nursing and Midwifery, College of Health and Medical Science, Haramaya University, Harar, Ethiopia; 4 Clinical Trial Unit, Jimma University, Jimma, Ethiopia; Debre Tabor University, ETHIOPIA

## Abstract

**Background:**

Anemia in school children is a worldwide public health problem, affecting about a quarter of this population. It also remains a significant problem in developing countries, with multifactorial causes. Anemia in school children has adverse effects on the development of the physical, cognitive, immunity of affected children, and subsequently their educational achievement which may lead to loss of productivity at a later age in life. Regular surveillance that could provide evidence-based local data is required to intervene in the problems. Therefore, this study aimed to determine the prevalence and associated factors of anemia among school children in primary schools of eastern Ethiopia.

**Methods:**

School-based cross-sectional study was conducted by recruiting 482 school- children. Data on socio-demographic and dietary habits were collected from parents/legal guardians. Capillary blood for blood film preparation and hemoglobin measurement and stool sample for the diagnosis of intestinal parasites infection was collected. Hemoglobin concentration was measured using a hemoglobinometer HemoCue^®^ 301+, and stool examination by direct wet mount and concentration technique. Data were entered into epi-data and exported into SPSS for analysis. Bivariate and multivariate logistic regression was run to identify associated factors. Association was described using adjusted OR (AOR) along with 95% CI and variables with a p-value<0.05 were considered statistically significant.

**Results:**

The overall prevalence of anemia was 24.5%. Being female (AOR = 2.88, 95% CI: 1.69, 4.92), family size of more than 5 (AOR = 2.78, 95% CI: 1.60, 4.81), not consuming green leafy vegetables (AOR = 4.09, 95% CI: 2.42, 6.94), consumption of milk (AOR = 2.22, 95% CI: 1.27, 3.88), being stunting (AOR = 3.17, 95% CI: 1.70, 5.91) and parasite infections (AOR = 5.23, 95% CI: 2.77, 9.85) were significantly associated with anemia.

**Conclusion:**

In this study nearly one-fourth of children were anemic. Anemia was a moderate public health problem among schoolchildren in the study area. Thus, school-based interventions targeting nutritional factors and intestinal parasite infection need to be implemented.

## Introduction

Anemia is a significant worldwide public health problem affecting both developed and developing nations [1]. Globally, it affects about 1.74 billion (22.8%) of the world’s population [2] of which 305 million (25.4%) are school-age children. In Ethiopia, anemia remains a mild to severe public health problem with prevalence ranging from 7.6% [3] to 43.7% [4]. The finding of a Nationally conducted systematic review and meta-analysis indicated 23% of schoolchildren were anemic [5]. Anemia occurs at all ages, however; reproductive-age women, preschool, and school-age children are affected more [6]. School children are one of the most vulnerable groups because they are at the age where physical growth and physiological change are fast enough which leads to the high demand for nutritional requirements [7]. Moreover, they are more vulnerable to intestinal parasite infections which are a major contributor to anemia [8,9].

Anemia has significant adverse health consequences and impacts on social and economic development. Globally, it causes 68.36 and 58.6 million years lived with a disability in 2010 [10] and 2019 [2] respectively. Anemia harms the physical, and cognitive development of affected children and subsequently their educational achievement which may lead to loss of productivity at a later age in life [11]. It also affects the function of immune systems and consequently increases the risk of infections by impacting both humoral and cellular immunity [12,13].

There are multiple and complex contributing factors to anemia including nutritional deficiency (iron, folate, and vitamin B12), genetic hemoglobin disorder, intestinal parasitic infections, and acute and chronic infections [14]. Pathophysiology of anemia is diverse based on its underlying etiologies which may attribute to decreased production from micronutrient deficiency because of poor dietary diversity [15] and increased destruction and/or blood loss due to parasite infections [16]. Generally, anemia is not a disease, but instead the manifestation of an underlying pathological process that occurs due to multiple contributing factors. Given the multifactorial nature, control, and prevention of anemia require an integrated approach based on the identification and addressing of specific risk factors. This requires regular surveillance that could provide evidence-based local data to policymakers to design plans and policies to intervene in the problems. Although the previous study in the local area assessed the magnitude of anemia with association to other factors, intestinal parasites, and nutritional status were not considered and the morphological type of anemia was not assessed. Therefore, this study aimed to assess the current magnitude, morphological type, and associated factors of anemia among school- children in Kersa Woreda primary school, eastern Ethiopia.

## Materials and methods

### Study setting and population

A school-based cross-sectional study was conducted among primary school children in Kersa woreda, eastern Ethiopia, from December 7, 2020, to January 7, 2021. Kersa Woreda is located 478 km to the east of Addis Ababa and 42 km to the west of Harar city. The Woreda is located at an altitude ranging from 1400 to 3200 m above sea level. There are 38 kebeles, of which 35 are rural and 3 are town kebeles. According to the 2007 population and housing census of Ethiopia, the projected total population of the Kersa district was 172,626, of whom 87,029 were men and 85,597 were women [17]. According to the Office of Education of Kersa Woreda, there are a total of 101 primary schools, of which 94 and 7 are public and private, respectively. Out of 101, 27 schools have all grade levels from 1–8. There are approximately 45597 primary school students. Primary school children aged 6–17 who were willing and whose parents gave consent were included in the study. On the other hand, children who had been receiving hematinic factors such as iron, vitamin B12, and foliate for three months and anti-helminthic medication two weeks before the study were excluded.

#### Sample size determination and sampling technique

**Fig 1:**
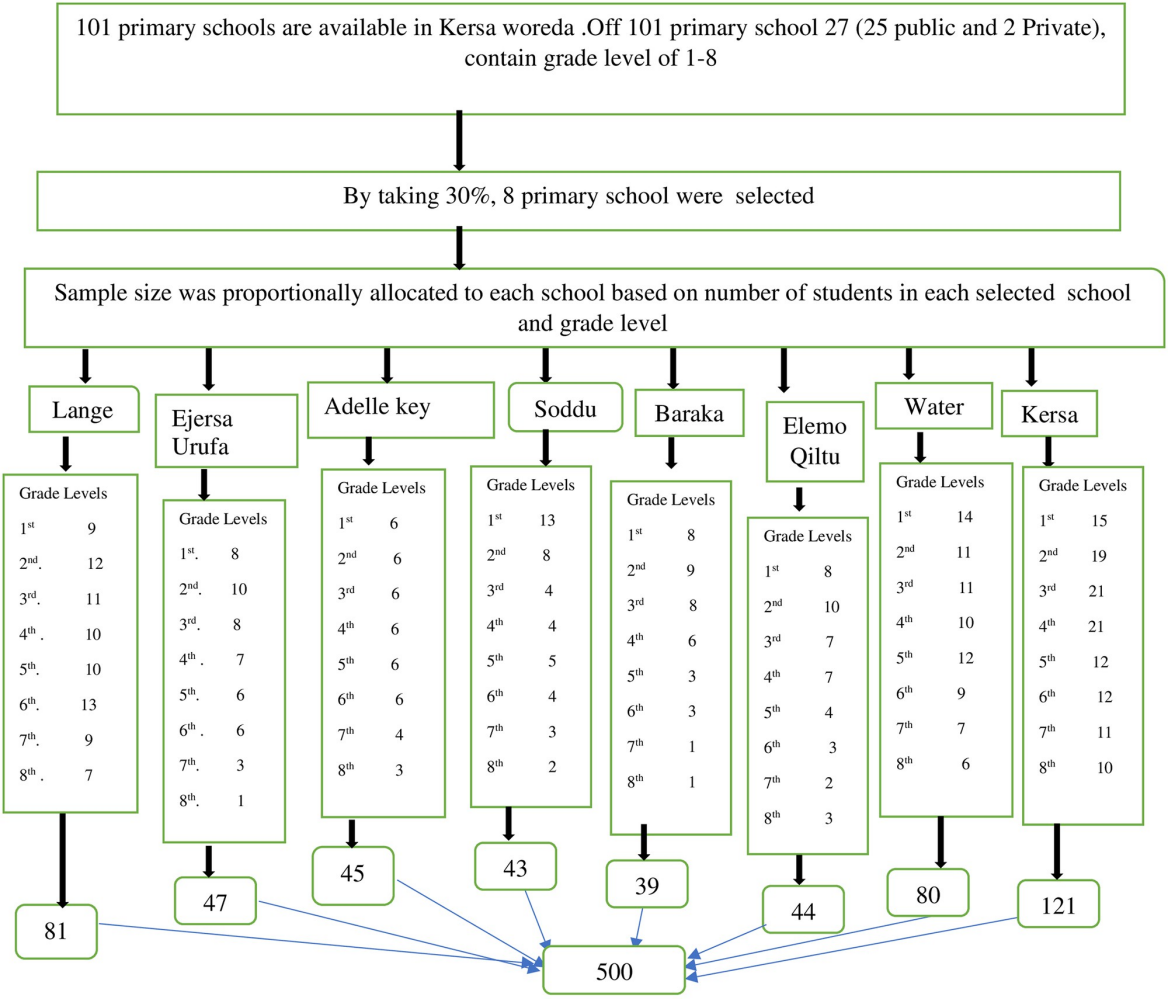
Schematic diagram showing sampling technique of school-children attending primary school in Kersa woreda, eastern Ethiopia, 2020/2021.

A total of 500 sample sizes was calculated using single population proportion statistical formula n = (Zα /2)^2^ p (1-p)/d^2^ by considering assumptions: 0.27 P (proportion) [18], 95% level of confidence, 5% margin of error, 1.5 design effect, and 10% non-response. The study participants were chosen using a multi-stage sampling method. First, 27 primary schools with grade levels 1 through 8 were found, and 30% of those were included. Then, eight primary schools were chosen using simple random sampling. The K-value was computed by dividing the total number of the study population by the entire sample size (K = 9651/500 = 19). The total sample size was proportionally allocated among the selected schools based on the number of students. The proportionally allocated sample size was further distributed to grade level considering the number of students (Fig 1). Finally, a systematic random sampling technique was used to select study participants, using each school student’s registration detail for an academic year as a sampling frame.

### Data collection tools and methods

#### Socio-demographic and related data

Schools were sampled and children were selected at school. Then, children’s parents/ legal guardians were contacted by Health Extension workers, and data on socio-demographic, and dietary habits were collected through face-to-face interviews using a semi-structured questionnaire that is extracted from different literature. Dietary factors were collected and assessed by using a modified version of the Helen Keller International Food Frequency Questionnaire (FFQ) that was used previously in Ethiopia [19]. Consumption of commonly consumed food items in the area was grouped into five as vegetables, fruit, meat, dairy product, and coffee/ tea was assessed.

#### Anthropometric measurement

Anthropometric measurements were taken according to WHO guidelines for anthropometric measurements [20]. Weight was measured to the nearest 0.1 kg using a portable digital weight measuring scale. Each child was weighed with light clothing and barefoot. The weighing scale was calibrated using the standard calibration weight of 2kg iron bars. Height was measured in the Frankfurt position using a locally manufactured stadiometer with a sliding head bar. All measurements were performed in duplicate and the average value was used for anthropometry data analysis. Then anthropometric measurements were converted into Height-for-Age Z scores (HAZ) and Body Mass Index for-Age Z scores (BAZ) using WHO Anthro Plus version 1.0.4.

#### Sample collection, processing, and analysis

Capillary blood samples for hemoglobin measurement and peripheral blood film preparation were collected from each study participant by finger prick using a sterile single-use disposable lancet. Hemoglobin concentration was measured using a portable digital hemoglobinometer (HemoCue^®^301+, Angel Holm, Sweden). After correcting for altitude, adjusted Hb concentration was used to define anemia. Blood films were prepared, stained with wright stain, and examined microscopically for evaluation of red cell morphology. Approximately 2 grams of a stool sample for parasite examination were collected following the standard procedures for stool sample collection. Collected samples were preserved using 10% formalin and intestinal parasites were examined by both direct wet mount and formol-ether concentration techniques.

### Operational definition

**Table:** 

Anemia is defined based on WHO recommendations for age and gender [21]				
**Age category**	**Non-anemic**	**Anemic**		
		Mild	Moderate	Severe
6–11 years	≥11.5 g/dl	11–11.4 g/dl	8–10.9 g/dl	<8g/dl
12–14 years	≥12 g/dl	11–11.9 g/dl	8–10.9 g/dl	<8g/dl
Male 15–17 years	≥13 g/dl	11–12.9 g/dl	8–10.9 g/dl	<8g/dl
Female 15–17 years	≥12 g/dl	11–11.9 g/dl	8–10.9 g/dl	8–10.9 g/dl
Underweight, stunting, and thinness are defined based on WHO growth reference for school-aged children and adolescents [22]				
**Underweight**	Z-score less than -2 SD from the median of the WHO growth reference for weight-for-age			
**Stunting**	Z-score less than -2 SD from the median of the WHO growth reference for height-for-age			
**Thinness:**	Z-score less than -2 SD from the median of the WHO growth reference for BMI for age [22]			
**School children**	children aged 6–17 years [3,5]			

#### Data quality assurance and quality control

To assure the quality of data, all phases of quality assurance were maintained. The questionnaire prepared in English was translated into the local language, Afaan Oromo. Pre-tested was conducted on 25 (5%) of the total sample size. The training was given to data collectors and supervisors for two consecutive days on the objective of the study, confidentiality of information, and the data collection process. Standard operating procedures for hemoglobin measurement, blood film preparation, staining [23], and formol-ether concentration technique [24] were strictly followed. All laboratory reagents were checked for their expiry date before use. The accuracy of HemoCue and micro cuvettes were checked by comparing Hb values with those measured on an automated hematology analyzer (UniCel DxH800 Beckman Coulter, USA) on 20 samples of patients at Hiwot Fana Specialized University Hospital. The quality of the Wright stain was checked on thin blood film stained at different staining times by comparing the staining characteristics of RBC and WBC. Laboratory results were properly recorded on report formats using participants’ identification numbers.

#### Data processing, analysis, and interpretation

All data were checked for completeness and coded. Data entry was done using Epi Data version 3.1 and exported into SPSS version 20 for analysis. Descriptive statistics were used to describe study variables. Both bivariable and multivariable binary logistic regression were computed to identify associated factors. Variables with a p-value <0.25 in bivariate analysis were considered a candidate for multivariable analysis. Multi-co-linearity was checked using the variance inflation factor (VIF) test and all candidate variables were included in the final model of multivariable analysis. The model goodness of fit was tested using the Hosmer-Lemeshow goodness of fit test (p = 0.46) Adjusted odds ratio (AOR) with the corresponding 95% confidence interval (CI) was used to indicate the strength of the association and a variable with a P-value <0.05 was considered statistically significant.

### Ethical consideration

Ethical clearance was obtained from Jimma University Institutional Review Board (Ref. No: IRB00937/2020). A letter of support was submitted to Kersa Woreda Health Bureau and Education office to obtain official permission and obtained official permission was submitted to each school director. Informed written consent from the parents/legal guardians of children and oral assent from children below the age of 18 years were obtained after describing the benefits and risks of the study. The following measures were taken to ensure confidentiality: Participant names and other identifiers were not used; only participant codes were used during data collection, entry, and analysis on the computer. The data was only accessible to authorized people, including the data collector and investigator. To protect their privacy, the children’s parents were also interviewed separately. All collected data were exclusively utilized for the study. Children who tested anemic and positive for intestinal parasites were connected to nearby health institutions for proper treatment.

## Results

### Socio-demographic characteristics

Out of the total sample size (500) school children recruited for the study, 482 were enrolled in this study with a response rate of 96.4%, and 7 children took anti-parasite medication in the last two weeks, 3 children with hematin factors treatment in the last three months before the study and 8 children who were not volunteer to give stool and blood sample were excluded.

The age of the study participant ranges from 6 to 17 years, with a median age of 10 years. More than half, 52.7% (n = 254) of study participants were male. About 57.7% (n = 278) of study participants were in the grade level of 1–4 and around 61% (n = 294) were rural residents. Regarding the occupational status of parents, the majority, 63.1% (n = 304) of children’s fathers were farmers, and most, 73% (n = 352) of the mother were housewives, whereas about 44.8% (n = 216) of child fathers and majority, 62.7% (n = 302) mothers are unable to read and write. About 46.7% (n = 225) of children’s parents have monthly household incomes <500 Ethiopian Birr and nearly more than half have a family size of more than five (Table 1).

**Table 1:** Sociodemographic characteristics of parents/legal guardians and school children in Kersa Woreda primary schools, eastern Ethiopia, 2020/21(n = 482).

Variable	Category	Frequency	Percentage
Age (in years)	6–9	207	42.9
	10–14	219	45.4
	15–17	56	11.6
Sex	Male	254	52.7
	Female	228	47.3
Students Grade Level	1–4	278	57.7
	5–8	204	42.3
Residence	Rural	294	61
	Urban	188	39
Father’s occupation	Farmer	304	63.1
	Merchant	35	7.3
	Private employee	33	6.8
	Government employee	110	22.8
Mothers Occupation	Housewife	352	73
	Merchant	51	10.6
	Private employee	17	3.5
	Government employee	62	12.9
Fathers educational status	unable to read and write	216	44.8
	Primary education	104	21.6
	Secondary and above	162	33.6
Mothers educational status	unable to read and write	302	62.7
	Primary education	76	15.8
	Secondary and above	104	21.6
Average monthly income in Ethiopia birr	<500	225	46.7
	500–1999	66	17.8
	≥2000	171	35.5
Family Size	≤5	233	48.3
	>5	249	51.7

### Dietary factors

Food consumption is classified based on reviewed previous literature [25–27]. About one-third, 33.2% (n = 160) of study participants responded not to have access to green leafy vegetables, while about 47.8% (n = 154) of consumers had access more than once a week. Of the total study participants, nearly more than half, 55.2% (n = 266) of children consumed milk, and the majority, 64.5% (n = 311) of them had a habit to take coffee/tea after the meal (Table 2).

**Table 2:** Dietary factors of school-children in Kersa Woreda primary schools, eastern Ethiopia, 2020/21 (n = 482).

Variable	Category	Frequency	Percentage
Consumption of green leafy vegetables	Yes	322	66.8
	No	160	33.2
Frequency of green leafy vegetable consumption	Daily	93	28.9
	Once a week	75	23.3
	More than once a week	154	47.8
Consumption of citrus fruit	Yes	195	40.5
	No	287	59.5
Frequency of citrus fruit consumption	Daily	8	4.1
	Once a week	168	86.2
	More than once a week	19	9.7
Consumption of red meat	Yes	197	40.9
	No	285	59.1
Frequency of red meat consumption	Once a week	161	81.7
	More than once a week	36	18.3
Consumption of milk	Yes	266	55.2
	No	216	44.8
Frequency of Milk consumption	Daily	58	21.8
	More than once a week	136	51.1
	Once a week	72	27.1
Consumption of coffee/tea after a meal	Yes	311	64.5
	No	171	35.5

### Nutritional status and clinical factors

Of a total of study participants, about 16% (n = 77) were stunted for their age (HAZ <- 2 SD) and 11.8% (n = 57) were thin for their age (BAZ<-2 SD). Among study participants examined for intestinal parasite infection, 16.2% (n = 78) were infected with at least one intestinal parasite. A total of six species of intestinal parasites were identified. *Schistosoma mansoni* 28(16.2%), *Giardia lamblia* 22 (4.6%), and *Haymenolepsis nana* 13(2.7%) were predominant parasites. In addition to this, two cases with double infection (*Giardia lamblia and Haymenolepsis nana*) and one with triple infection (*Ascaris lumbricoid*, *Schistosoma mansoni*, *and Giardia lamblia*) were also identified (Table 3).

**Table 3:** Nutritional status and clinical factors of school-children in Kersa Woreda primary schools, eastern Ethiopia, 2020/21 (n = 482).

Variable	Frequency	Percentage
Stunting (HAZ <-2SD)		
Yes	77	16
No	405	84
Thinness (BAZ<-2 SD)		
Yes	57	11.8
No	425	88.2
Intestinal parasite infection		
Yes	78	16.2
No	404	83.8
Types of Intestinal parasite		
*S*. *mansoni*	28	16.2%
*G*.*lamblia*	22	4.6%
*H*. *nana*	13	2.7%
*A*.*lumbaricoids*	4	
Hookworm	6	
*E*.*histolytica*/dispar	5	

### Prevalence, severity, and types of anemia

The mean hemoglobin value of school- children was 13.4±1.83g/dl, ranging from 6.3–17.8 g/dl. The overall prevalence of anemia was 24.5% (118/482 95% CI: 20.6–28.6) and high prevalence was detected in the females and age group of 10–14 years, 58/118 (49.2%) followed by age 5–9, 44/118 (37.3%) and 15–17 years, 16/118 (13.6%). Among those who were anemic, 65 (55.1%) (95% CI: 45.8–63.6) had mild, 49 (41.5%) (95% CI: 33.1–50.8) had moderate anemia, and four cases of severe anemia were also identified. Regarding types of anemia, the examined blood film showed 68 (57.6%) microcytic hypochromic, 28 (23.7%) normocytic normochromic, and 22 (18.6%) macrocytic normochromic cells.

### Factors associated with anemia

Independent variables including Sex (being female) (AOR = 2.88 95% CI: 1.69, 4.92), family size of more than five (AOR = 2.78, 95% CI: 1.60, 4.81), non-consumption of green leafy vegetables (AOR = 4.09, 95% CI: 2.42, 6.94), consumption of milk (AOR = 2.22, 95% CI: 1.27, 3.88), nutritional status (being stunted) (AOR = 3.17, 95% CI: 1.70, 5.91) and being positive for intestinal parasite infection (AOR = 5.23, 95% CI: 2.77, 9.85) were remain as independent predictors of anemia in the final model of multivariate analysis (p<0.05) (Table 4).

**Table 4:** Factors associated with anemia among school-children in Kersa Woreda primary schools, eastern Ethiopia, 2020/21 (n = 482).

Variable	Category	Anemia		COR (95% CI)	AOR (95% CI)
		Anemic	Non-Anemic		
Sex:	Female	76 (33.3%)	152 (66.7%)	2.52 (1.64,3.88)	**2.88 (1.69, 4.92)** **
	Male	42 (16.5%)	212 (83.5%)	1	1
Mother-	Unable to read and	90 (29.8%)	212 (70.2%)	1.839(0.95, 3.51)	1.14 (0.58, 2.26)
	write Primary	11(14.5%)	65 (85.5%)	0.28 (0.30, 1.76)	0.34 (0.125, 0.92)
	Secondary and above	17 (16.3%)	87 (83.7%)	1	1
Family size	>5	88 (35.3%)	161 (64.7%)	3.7 (2.33, 5.88)	**2.78 (1.603, 4.81)** **
	≤5	30 (12.9%)	203 (87.1%)	1	1
Consumption of green leafy veg.	No	73 (45.6%)	87 (54.4%)	5.17 (3.32,8.04)	**4.09 (2.42, 6.94)** **
	Yes	45 (14%)	277 (86%)	1	**1**
Consumption of citrus fruit	No	83 (28.9%)	204 (71.1%)	1.86 (1.19,2.91)	1.52 (0.86, 2.70)
	Yes	35 (18%)	160 (82%)	1	1
Consumption of red meat	No	87 (30.5%)	198 (69.5%)	2.35 (1.49,3.72)	1.45 (0.74, 2.82)
	Yes	31 (15.7%)	166 (84.3%)	1	1
Consumption of milk	Yes	89 (33.5%)	177 (66.5%)	3.24 (2.03, 5.17)	**2.22 (1.27, 3.88)** *
	No	29 (13.4%)	187 (86.6%)	1	1
Consumption of coffee/ tea	Yes	87 (28%)	224 (72%)	1.75 (1.11,2.78)	1.40 (0.80, 2.46)
	No	31 (18.1%)	140 (81.9%)	1	1
Stunting	Yes	41 (53.25%)	36 (46.75%)	4.85 (2.91,8.09)	**3.17 (1.70, 5.91)** **
	No	77 (19%)	328 (81%)	1	1
Thinness	Yes	18 (31.6%)	39 (68.4%)	1.5 (0.82, 2.74)	1.762 (0.84, 3.71)
	No	100 (23.5%)	325 (76.5%)	1	1
IPI	Yes	48 (61.5%)	30 (38.5%)	7.67 (4.49,13.08)	**5.23 (2.77, 9.85)** **
	No	70 (17.3%)	334 (82.7%)	1	1

* p<0.05,

**p<0.001,

CI = Confidence interval, COR = Crude OR, AOR = Adjusted OR, IPI = Intestinal parasite infection.

## Discussion

The overall prevalence of anemia among schoolchildren was 24.5%, indicating a moderate public health problem according to the WHO classification of anemia as a problem of public health significance [28]. The prevalence of anemia found in this study is comparable to that seen in Angola, 21.6% [29], Cape Verde, 23.8% [30], and two studies reported in Ethiopia [31,32]. Nonetheless, higher than the results from research conducted in Northeastern Brazil, 9.3% [33], Vietnam, 12.9% [34], Cameron, 5% [35], and other parts of Ethiopia [27,36,37]. This could be due to variations in the geographical area, study setting, socioeconomic status, and age group included in the study. This study primarily concerns children who are attending schools in predominantly rural areas, while studies in Durbete, Bonga, Jimma, and Gondar Town focus on those individuals who are found in urban areas. In this study, we considered a broad age range of 6–17 years. This may be accounted for by the high prevalence of anemia in our findings as compared to the lower frequency observed in other studies with comparable designs.

In contrast, it is lower than the findings reported from Northwestern Nigeria, 40.3% [38], Egypt, 59.3% [39], and five studies conducted in various sections of Ethiopia [25,26,40–42]. The discrepancy might be due to differences in the study setting, sample size, and epidemiological distribution of parasite infection. This study is schools based whereas a study that was carried out in the Arba Minch area, Pawe, and Jimma town was community-based. Another explanation for this variance in magnitude could be the epidemiological spread of intestinal parasite infection. According to a study from northwest Nigeria, hookworm was the most common parasite and 53% of infected children had anemia [38]. Studies in other parts of Ethiopia also indicated that anemia is substantially associated with intestinal parasites, where the incidence of these parasites ranges from 30 to 46.5%, with hookworm being the most common and contributing significantly to anemia [26,40,42].

The prevalence of anemia in this study was significantly associated with being female, family size of more than five, non-consumption of green leafy vegetables, consumption of milk, stunting, and intestinal parasite infection. The likelihood of anemia in females was 2.88 (AOR = 2.88) times higher as compared to males. This finding is in line with the study reported from Egypt [39] and southwest Ethiopia [27]. This is might be related to the combined effect of both rapid physical growth and the occurrence of menarche in adolescent girls, which might make females at the most risk of anemia. In addition to this, a high prevalence of anemia is detected in females and the age group 10–14 years in our finding which might be an early age of menarche and the production of estrogen hormone in females. Studies indicated that the occurrence of menarche significantly increase female’s iron requirement due blood loss [43,44], whereas the estrogen hormone has an antagonist effect on erythropoiesis. This condition with other factors may put females at more risk of anemia. On the other hand, male at the age of puberty starts to produce testosterone hormone having an agonist effect on erythropoiesis and this condition may put them at less risk of anemia. Studies suggested that testosterone increases erythropoiesis via increased erythropoietin [45,46].

The chance of being anemic among children from a family size of more than five was 2.78 (AOR = 2.78) times compared to those with a family size of less than five. A similar finding was reported from a study done in northwest Ethiopia [47], and southwest Ethiopia [27] which showed that children from large family sizes were more likely to be anemic than those from low family sizes.

The risk of having anemia was 4.09 (AOR = 4.09) times higher among children who did not consume green leafy vegetables as compared to the consumer. This is consistent with the study conducted in Jimma town [25,42] which reported that children who consume food from plant sources less are more likely to be anemic. This suggested that limited access to green leafy vegetables which are rich in micronutrients may primarily lead to anemia. The green leafy vegetable is a rich source of micronutrients such as iron and vitamins [48]. Unfortunately, the presence of antinutritional factors such as phytate and polyphenol acts to reduce the bioavailability of iron [49]. However, heat cooking has been shown to reduce the level of antinutritional factors in vegetables and increase the bioavailability of iron [50].

The odds of anemia in children who consumed milk were 2.22 (AOR = 2.22) times higher as compared to non-consumer. The possible justification for this might be the inhibitory effect of mineral calcium and protein found in milk on iron absorption in the absence of iron absorption enhancers [51]. There is scientific evidence that suggested mineral calcium and protein found in dairy products affect the absorption of iron in the diet. Calcium inhibits iron absorption by blocking the divalent metal transporter-I (DMT-I) uptake of iron [52,53]. Similarly, Casein protein binds iron with high affinity and forms a complex, and makes iron non-absorbable [54]. Consumption of food rich in vitamin c such as fruit and green leafy vegetable is advantageous to enhancing iron absorption.

The other variable significantly associated with anemia was the nutritional status of children. Stunted children were 3.17 (AOR = 3.17) times more likely to be anemic. This finding is consistent with the finding of previous studies which showed that stunted children are more at risk of anemia than non-stunted children [32,37,40]. This might be due to inadequate intake of a diversified diet and/or increased nutrient loss due to intestinal parasite infection. Addressing the nutritional issue of school children through implementing a program like a school-based feeding practice might be very important to improve the nutritional status and prevent the continuation of stunting. Community education to create awareness on adequate dietary intake and prevention of intestinal parasites is also important.

School children who were positive for intestinal parasite infection were, 5.23 (AOR = 5.23, 95% CI: 2.77, 9.85) times more likely to be anemic than non-infected children. Comparable with the studies conducted in Northwestern Nigeria [38], Egypt [39], and Ethiopia [25,26,32,37,40] which indicated intestinal parasite infection as one of the factors that increase the risk of anemia. This could be explained by the contribution of the identified intestinal parasites to anemia through different mechanisms which could be blood loss, impaired nutrient absorption, reduction in nutrient intake due to induced loss of appetite, autoimmune hemolysis, and inflammation [55–57]. Intervention approaches including periodical school-based deworming, health education, enhancing sanitation services, and encouraging methods to maintain personal hygiene will help to control the extent of intestinal parasite infection [58,59].

This study is interpreted with some strengths and limitations. It provides information on the current prevalence and identifies associated factors of anemia among school children in the study area. In addition to the measurement of hemoglobin concentration, we try to assess the morphological type of anemia through a detailed morphological examination of a red blood cell. Additionally, an adjustment of hemoglobin for variation of altitude was made to avoid underestimating the prevalence of anemia. Despite this, the study has some limitations. One limitation is that we didn’t measure micronutrients like serum ferritin, folate, and vitamin B12 to identify the specific cause of anemia. The other limitation is the cross-sectional nature of the study design which makes the inference impossible to determine a cause-effect relationship.

## Conclusion and recommendation

Nearly one-fourth of the schoolchildren in the study area were anemic. Females and children aged 10 to 14 years were more likely to be anemic. More than half of anemic children had mild anemia. Overall, anemia was a moderate public health problem among schoolchildren in the study area. Being female, having a family size greater than five, a non-consumption of green leafy vegetables, consuming milk, stunting, and intestinal parasite infection was significantly associated with anemia. Thus, school-based interventions targeting intestinal parasites and nutritional factors need to be implemented. To minimize the risk of intestinal parasite infection, health education to promote personal hygiene and environmental sanitation should be given. In addition, school-based regular deworming should be provided to students. Setting policy and implementing a program like school-based feeding practices might be very important to improving the nutritional status of schoolchildren.

## Supporting information

S1 File "Data set used for analysis" includes data on anemia and associated factors. (XLSX)

## Abbreviation

BAZ: Body Mass Index-for-Age Z score
DALYs: Disability Adjusted Life Years
HAZ: Height-for-Age Z scores
Hb: Hemoglobin
RBC: Red Blood Cell
WAZ: Weight-for-Age Z scores
WHO: World Health Organization
